# Construction and efficacy evaluation of an early warning scoring system for septic shock in patients with digestive tract perforation: A retrospective cohort study

**DOI:** 10.3389/fmed.2022.976963

**Published:** 2022-09-13

**Authors:** Peiling Chen, Jingqi Gao, Jun Li, Rongguo Yu, Ling Wang, Fangqin Xue, Xiaochun Zheng, Ling Gao, Xiuling Shang

**Affiliations:** ^1^Shengli Clinical Medical College of Fujian Medical University, Fuzhou, China; ^2^The Third Department of Critical Care Medicine, Shengli Clinical Medical College of Fujian Medical University, Fujian Provincial Hospital, Fujian Provincial Center for Critical Care Medicine, Fujian Provincial Key Laboratory of Critical Care Medicine, Fuzhou, China; ^3^Department of Pharmacy, Shengli Clinical Medical College of Fujian Medical University, Fujian Provincial Hospital, Fuzhou, China; ^4^Department of Gastrointestinal Surgery, Shengli Clinical Medical College of Fujian Medical University, Fujian Provincial Hospital, Fuzhou, China; ^5^Department of Anesthesiology, Shengli Clinical Medical College of Fujian Medical University, Fujian Provincial Hospital, Fujian Emergency Medical Center, Fujian Provincial Key Laboratory of Critical Care Medicine, Fujian Provincial Co-constructed Laboratory of “Belt and Road,” Fuzhou, China

**Keywords:** septic shock, gastrointestinal tract, perforation, risk factors, early warning score

## Abstract

**Objective:**

To establish an early warning scoring system for septic shock in patients with digestive tract perforation (DTP) and evaluate its diagnostic efficacy.

**Methods:**

Patients with surgically confirmed or clinically diagnosed DTP admitted to the Department of Intensive Care Medicine of Fujian Provincial Hospital from June 2012 to October 2021 were retrospectively analyzed. General demographic characteristics, perforation-related information, vital signs, common laboratory indicators, and common ICU scores (Glasgow Coma Scale score, Acute Physiology and Chronic Health Evaluation-II score,Sequential Organ Failure Assessment score) were collected. The patients were divided into shock group and non-shock group according to whether the patients had septic shock during hospitalization. The risk factors of septic shock were screened by basic statistical analysis and multivariate Logistic regression analysis. The receiver operating characteristic curve was drawn to determine the cut-off value of the continuous indicators and discretized with reference to clinic, and the corresponding score was set according to the β regression coefficient of each variable.

**Results:**

A total of 176 patients with DTP were included. The average age of the patients was 64.13 ± 14.67 years old, and 74.40% were males. The incidence of septic shock was 30.11% (53/176). Multivariate Logistic regression analysis showed that the highest heart rate≥105 beats/min, Glasgow Coma Scale score≤14 points, lactic acid≥5.75 mmol/L, procalcitonin≥41.47 ug/L, C-reactive protein≥222.5 mg/L were independent risk factors for septic shock in patients with DTP. The total score of clinical diagnostic scoring system of septic shock in patients with DTP was 6 points, including the highest heart rate≥105 beats/min (1 point), lactic acid≥5.75 mmol/L (two points), procalcitonin≥41.47 ug/L (one point), C-reactive protein≥222.5 mg/L (1 point), and Glasgow Coma Scale score≤14 points (1 point). The area under ROC curve (AUC) of this scoring system was 0.789 and the 95% confidence interval was 0.717–0.860 (*P* < 0.001); when the optimal cut-off value was 2.5, the sensitivity and specificity were 54.70 and 87.80%, respectively.

**Conclusion:**

This new score system has its certain clinical value and has important guiding significance for clinicians to judge the prognosis of patients with DTP in time.

## Background

Digestive tract perforation (DTP) is a potentially devastating complication that may result from various disease processes and is an important indication of emergency surgery. The most common conditions that cause gastrointestinal perforation are peptic ulcer, gastrointestinal tumor, trauma, and inflammatory bowel disease. If left untreated, it leads to death. Although the incidence of DTP has decreased significantly over the past 30 years, especially due to the development of intensive care technology, the advancement of treatment concepts, and the development of various new drugs, the mortality rate is still high (1, 2). According to statistics, the average 30-day mortality rate of peptic ulcer perforation is 23.75% (3). Another report pointed out that gastrointestinal perforation accounted for 40% of peptic ulcer-related deaths, and its 90-day mortality rate was as high as 30% (4–6). Multiple factors, including advanced age, use of non-steroidal anti-inflammatory drugs, diabetes, and use of glucocorticoids, have been associated with increased mortality in patients with DTP (7–10).

Septic shock is a major risk factor for increased mortality in patients with DTP (2, 4). Five studies in Europe, Asia, and Africa reported a significant increase in shock-related mortality in patients with DTP (11–15). Also, septic shock after DTP is a common critical illness in intensive care units (ICU) (16). It is estimated that 30–35% of patients with DTP have sepsis before they arrive in the operating room, and 25% of patients develop septic shock within 30 days of surgery (17).

Recent studies indicated that early recognition and appropriate management of the first few hours after septic shock could significantly improve the prognosis of patients (18). However, so far, no early identification methods have been proposed for screening patients with DTP. Therefore, based on quantitative clinical data, in this study, we analyzed risk factors of septic shock in the patients with DTP and established an early warning scoring system for septic shock in patients with gastrointestinal perforation, aiming to assist clinicians in early identification and intervention of patients with DTP, so as to reduce the occurrence of adverse outcomes.

## Materials and methods

### Study design

Patients with surgically confirmed or clinically diagnosed DTP admitted to the Department of Intensive Care Medicine of Fujian Provincial Hospital from June 2012 to October 2021 were retrospectively analyzed. DTP was defined as the destruction of the integrity of the digestive tract, i.e., complete non-invasive penetration of the wall of the esophagus, stomach, small intestine, or large intestine (19). The clinical diagnostic criteria were the presence of free gas under the diaphragm by the plain abdominal film in a vertical position, or the presence of gas-liquid coexistence by abdominal ultrasound, or the presence of free gas in the abdominal cavity by abdominal computed tomography (CT) (20, 21). The diagnostic criteria for septic shock were in line with sepsis-3.0 (22), i.e., patients with sepsis had persistent hypotension after adequate volume resuscitation and needed vasoconstrictor drugs to maintain mean arterial pressure (MAP) ≥ 65 mmHg and serum lactate level > 2 mmol/L.

Patients were divided into shock group and non-shock group according to whether septic shock occurred during hospitalization. Clinical data, including demographic characteristics, perforation-related information, vital signs, common laboratory indicators, ICU common scores (Glasgow Coma Scale score, GCS score;Acute Physiology and Chronic Health Evaluation-II score,APACHE-II score;Sequential Organ Failure Assessment score,SOFA score), and mortality rate within 28 days were collected and compared.

The study protocol was approved by the ethics committee (K2021-09-043), and since it was a retrospective study, the informed consent of patients was exempted from ethical approval.

### Research subjects

Inclusion criteria were: (1) patients admitted to the Intensive Care Unit of our Hospital from June 2012 to October 2021; (2) patient aged > 18 years at the date of admission; (3) patients with surgically confirmed or clinically diagnosed DTP based on the above criteria (see section Study design). Exclusion criteria were: (1) patients aged <18 years; (2) those with missing electronic medical records. All selected patients were routinely treated by the same associate chief physician with 10 years of experience in the field.

### Data collection

The general data of patients were collected, including gender, age, past medical history, perforation area, and perforation-operation time interval. Within 24 h of admission to ICU, the heart rate (HR), respiratory rate (RR), mean arterial pressure (MAP), oxygenation index (PaO2/FiO2), 24-h urine volume, serum sodium (Na^+^), serum potassium (K^+^), total bilirubin (TBIL), serum creatinine (SCr), platelet count (PLT), albumin (Alb), procalcitonin (PCT), C-reactive protein (CRP), the potential of hydrogen (pH), lactic acid (Lac), GCS score, APACHE-II score, SOFA score and the mortality rate within 28 days were collected and analyzed.

### Statistical analysis

Data were analyzed using SPSS 25.0 statistical software. Quantitative data conforming to a normal distribution were expressed as mean ± standard deviation (x̄± s), and the unpaired *t*-test was used for comparison between groups. The quantitative data with skewness distribution were expressed as median (quartile) [M (QL, QU)], and the Wilcoxon Mann-Whitney test was used for comparison between groups. Categorical data were expressed as percentages, and the chi-square test was used for comparison. Variables with *P* ≤ 0.05 (bilateral) were considered to be statistically significant and variables with *P* > 0.05 were excluded. The receiver operator characteristic curve (ROC curve) was used to analyze the retained continuous indicators to determine the cutoff value and were discretized into discrete indicators by referring to clinic. Taking the occurrence of septic shock as the dependent variable, the independent variables were screened by the method of forwarding stepwise regression (LR), and the independent risk factors of shock in patients with DTP were determined by multivariate Logistic regression analysis. Relative risk was expressed by odds ratio (OR) and 95% confidence interval (95%CI). The corresponding score was set according to the β coefficient of each risk factor, and the sum of each risk factor's scores was the patient's total risk score. The diagnostic efficiency of the scoring system was evaluated by area under ROC Curve (AUC). AUC ranged from 0.5 to 1.0, with <0.7 indicating low diagnostic value, 0.7–0.9 indicating moderate diagnostic value, and >0.9 indicating high diagnostic value.

## Results

### Characteristics of patients

**Figure 1:**
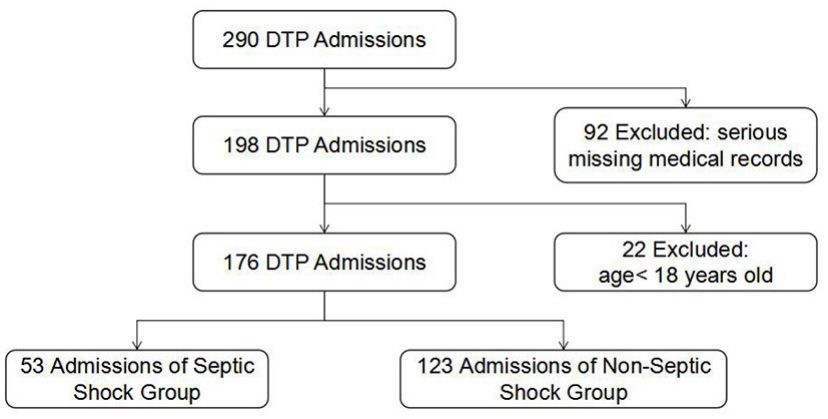
Flowchart of DTP cohorts analyzed showing the inclusion and exclusion criteria. DTP, digestive tract perforation.

A total of 290 patients with gastrointestinal perforation were screened. Then, 22 patients who were <18 years old and 92 patients with serious missing electronic medical records were excluded (Figure 1). Finally, 176 patients were included in the analysis, including 53 patients with septic shock and 123 patients without septic shock (Table 1). The average age of the patients was 64.13 ± 14.67 years, and 74.40% were male. The median perforation area was 2.13 (0.64, 3.09) cm^2^, and the median time interval from perforation to operation was 4.5(2.37, 53.02) h. The mortality rate within 28 days was 11.40%. APACHE-II score, SOFA score, highest HR, highest K^+^, Lac, PCT, CRP, and SCr in the shock group were higher than those in the non-shock group, while GCS score, Alb, pH, PaO2/FiO2 were lower than those in the non-shock group (all *P* < 0.05). However, there were no significant differences in gender, age, perforation area, the time interval from perforation to operation, 24-h urine volume, PLT, TBIL, and other indicators between the two groups.

**Table 1:** Comparison of general data of the two groups of patients.

**Item**	**Total** **(*n =* 176)**	**Shock group** **(*n =* 53)**	**Non-shock group** **(*n =* 123)**	**T/x^2^/z value**	***P*-value**
Age (years,x̄±s)	64.13 ± 14.67	64.08 ± 16.61	64.15 ± 13.83	0.33	0.974
Male [cases (%)]	131 (74.40%)	37 (69.80%)	94 (76.40%)	0.85	0.356
Perforation area [cm^2^,M(QL, QU)]	2.13 (0.64, 3.09)	2.25 (1.00, 3.09)	1.50 (0.64, 3.09)	0.84	0.399
Time interval from perforation to operation [h,M(QL, QU)]	4.5 (2.37, 53.02)	3.49 (2.45, 53.02)	5.05(2.19,53.02)	0.12	0.906
APACHE-II score (points, x̄±s)	17.30 ± 7.64	21.18 ± 7.69	15.63 ± 7.02	4.67	<0.001
SOFA score (points,					
=x±s)	8.68 ± 3.65	10.93 ± 3.13	7.71 ± 3.43	5.87	<0.001
GCS score (points,x̄±s)	10.50 ± 4.41	9.37 ± 4.24	10.99 ± 4.40	2.24	0.026
Highest HR (beats/min,x̄±s)	103.46 ± 22.29	111.85 ± 25.93	99.85 ± 19.55	3.02	0.003
Fastest RR (times/min, x̄±s)	22.01 ± 5.59	23.02 ± 7.21	21.58 ± 4.70	1.34	0.185
Minimum MAP (mmHg, x̄±s)	75.17 ± 16.58	72.50 ± 16.664	76.33 ± 16.48	1.41	0.161
24-h urine volume (ml, x̄±s)	1,418.55 ± 979.06	1,308.93 ± 1,103.11	1465.78 ± 921.34	0.98	0.331
Highest Na^+^ (mmol/L, x̄±s)	141.09 ± 4.97	141.8 ± 4.60	140.78 ± 5.11	1.25	0.215
Minimum Na+ (mmol/L, x̄±s)	139.36 ± 4.87	139.52 ± 4.28	139.29 ± 5.12	0.28	0.781
Highest K+ (mmol/L, x̄±s)	4.34 ± 0.58	4.51 ± 0.61	4.26 ± 0.56	2.62	0.010
Minimum K+ (mmol/L, x̄±s)	4.06 ± 0.53	4.12 ± 0.61	4.04 ± 0.50	0.97	0.335
PLT (*10^9^, x̄±s)	173.79 ± 91.10	163.21 ± 101.19	178.35 ± 86.43	1.01	0.313
Alb (g/L, x̄±s)	20.94 ± 6.54	18.96 ± 6.18	21.79 ± 6.52	2.69	0.008
PaO2/FiO2 (mmHg, x̄±s)	233.72 ± 114.03	204.5 ± 115.19	246.32 ± 111.65	2.26	0.025
pH [M(QL, QU)]	7.31 (7.29, 7.40)	7.30 (7.23, 7.38)	7.33 (7.30, 7.41)	2.77	0.006
Lac [mmol/L,M(QL, QU)]	3.65 (2.10, 4.65)	4.30 (2.45, 4.77)	3.20 (1.90, 4.65)	2.07	0.039
TBIL [umol/L,M(QL, QU)]	17.40 (11.10, 23.77)	17.22 (10.79, 24.46)	17.58 (11.31, 23.80)	0.16	0.877
SCr [umol/L,M(QL, QU)]	101.00 (74.25, 188.50)	149.47 (84.00, 223.00)	92.00 (71.00, 151.00)	3.11	0.002
PCT [ug/L,M(QL, QU)]	37.27 (7.82, 56.07)	45.41(24.26, 109.30)	27.85 (5.64, 45.41)	3.79	<0.001
CRP (mg/L, x̄±s)	172.78 ± 77.02	200.11 ± 80.94	161 ± 72.48	3.17	0.002
Mortality [cases (%)]	20(11.40%)	11(20.80%)	9(7.30%)	6.640	0.01

APACHE-II, acute physiology and chronic health evaluation II; SOFA, sequential organ failure assessment; GCS, Glasgow Coma Scale; HR, heart rate; RR, respiratory rate; MAP, mean arterial pressure; Na, sodium ion; K, potassium ion; PLT, platelet count; Alb, albumin; PaO2/FiO2, oxygenation index; Lac, blood lactate; TBIL, total bilirubin; SCr, serum creatinine; PCT, procalcitonin; CRP, C-reaction protein; 1 mmHg=0.13 3kPa.

### The optimal cut-off value of the continuous index is determined and discretized

ROC curve was used to analyze the continuous indicators, including highest HR, GCS score, Lac, SCr, PCT, CRP, highest K^+^, Alb, pH, SOFA score, and APACHE-II score (Table 2). The cut-off value corresponding to the maximum value of the Jorden index was taken as the diagnostic cut-off point to determine the optimal cut-off value (highest HR: 105 beats/min, Lac: 5.75 mmol/L, SCr: 116.5 umol/L, PCT: 41.47 ug/L, CRP: 222.5 mg/L, highest K+: 4.35 mmol/L, Alb: 18.15 g/L, pH: 7.28, PaO2/FiO2: 171.75 mmHg, GCS score: 14.5 points, SOFA score: 8.88 points, APACHE -II score: 22.5 points), and transformed into dichotomous data according to clinic.

**Table 2:** The optimal cut-off value and assignment of continuous indicators.

**Indicator**	**Optimal cut-off**	**Assignment**
Highest HR	≥105 beats/min	≥105 beats/min *=* 1,<105 beats/mi*n =* 0
Lac	≥5.75 mmol/L	≥5.75 mmol/L = 1,<5.75 mmol/L=0
SCr	≥116.5 umol/L	≥116.5 umol/L = 1,<116.5 umol/L = 0
PCT	≥41.47 ug/L	≥41.47 ug/L= 1,<41.47 ug/L= 0
CRP	≥222.5 mg/L	≥222.5 mg/L = 1,<222.5 mg/L = 0
Highest K+	≥4.35 mmol/L	≥4.35 mmol/L = 1,<4.35 mmol/L = 0
Alb	≤18.15 g/L	≤18.15 g/L = 1,>18.15 g/L = 0
pH	≤7.28	≤7.28 = 1,>7.28 = 0
PaO2/FiO2	≤171.75 mmHg	≤171.75 mmHg=1,>171.75 mmHg =0
GCS score	≤14 points	≤14 points =1,>14 points=0
SOFA score	≥8.88 points	≥8.88 points =1,<8.88 points=0
APACHE-II score	≥22.5 points	≥22.5 points =1,<22.5 points=0

APACHE-II, acute physiology and chronic health evaluation II; SOFA, sequential organ failure assessment; GCS, Glasgow Coma Scale; HR, heart rate; K^+^, serum potassium ion concentration; Alb, albumin; Lac, blood lactate; SCr, serum creatinine; PCT, procalcitonin; CRP, C-reaction protein; 1 mmHg=0.133 kPa.

### Univariate logistic regression analysis was used to screen the risk factors of septic shock in patients with DTP

Univariate Logistic regression analysis was used to screen the risk factors affecting the occurrence of septic shock (Table 3): With the occurrence of septic shock as the factor variable, univariate Logistic regression analysis was performed on the above discrete indicators (highest HR ≥ 105 beats/min, GCS score ≤14 points, Lac ≥5.75 mmol/L, SCr ≥116.5 umol/L, PCT ≥ 41.47 ug/L, CRP ≥ 222.5 mg/L, highest K^+^ ≥ 4.35 mmol/L, Alb ≤ 18.15 g/L, pH ≤ 7.28,PaO2/FiO2 ≤ 171.75 mmHg, SOFA score ≥8.88 points, APACHE-II score ≥22.5 points), and the results were all statistically significant indicators (all *P* < 0.05).

**Table 3:** Univariate logistic regression analysis on the occurrence of septic shock.

**Variable**	**β value**	**S x̄**	**X2 value**	**OR value**	**95%CI**	***P*-value**
Highest HR ≥ 105 beats/min	1.238	0.368	11.332	3.447	1.677~7.085	0.001
Lac ≥ 5.75 mmol/L	1.631	0.538	9.171	5.107	1.778~14.673	0.002
SCr ≥ 116.5 umol/L	0.960	0.340	7.968	2.613	1.341~5.089	0.005
PCT ≥ 41.47 ug/L	1.319	0.352	14.033	3.739	1.875~7.456	<0.001
CRP ≥ 222.5 mg/L	1.248	0.388	10.368	3.484	1.630~7.450	0.001
Highest K^+^ ≥ 4.35 mmol/L	1.080	0.394	7.531	2.944	1.362~6.368	0.006
Alb ≤ 18.15 g/L	0.884	0.340	6.774	2.421	1.244~4.712	0.009
pH ≤ 7.28	1.357	0.374	13.157	3.885	1.866~8.087	<0.001
PaO2/FiO2 ≤ 171.75 mmHg	1.304	0.357	13.333	3.683	1.829~7.415	<0.001
GCS score ≤ 14 points	0.976	0.360	7.345	2.653	1.310~5.373	0.007
SOFA score ≥8.88 points	2.033	0.410	24.607	7.639	3.421~17.058	<0.001
APACHE-II score ≥22.5 points	1.711	0.385	19.781	5.534	2.604~11.763	<0.001

APACHE-II, acute physiology and chronic health evaluation II; SOFA, sequential organ failure assessment; GCS, Glasgow Coma Scale; HR, heart rate; K+, serum potassium ion concentration; Alb, albumin; Lac, blood lactate; SCr, serum creatinine; PCT, procalcitonin; CRP, C-reaction protein; 1 mmHg=0.133 kPa; OR, odds ratio; 95% CI was 95% confidence interval.

### Multivariate logistic regression analysis was used to screen the independent risk factors of septic shock in patients with DTP

With the occurrence of septic shock as the dependent variable, the above discrete indicators (highest HR ≥ 105 beats/min, GCS score≤14 points, Lac ≥ 5.75 mmol/L, SCr ≥ 116.5 umol/L, PCT ≥ 41.47 ug/L, CRP ≥ 222.5 mg/L, highest K^+^ ≥ 4.35 mmol/L, Alb ≤ 18.15 g/L, pH ≤ 7.28,PaO2/FiO2 ≤ 171.75 mmHg, SOFA score ≥8.88 points, APACHE-II score ≥22.5 points) were included in the multivariate Logistic regression equation for analysis (Table 4). The independent variables were screened by Forward stepwise regression (LR). Independent risk factors of septic shock were: highest HR ≥ 105 beats/min (odds ratio (OR) = 2.977, 95% confidence interval (95% CI) was 1.405~6.311, *P* = 0.004), GCS score ≤14 points (OR = 2.494, 95% CI was 1.127~5.522, *P* = 0.024), Lac ≥ 5.75 mmol/L (OR = 4.907, 95%CI was 1.490~16.165), PCT ≥ 41.47 ug/L (OR = 2.821, 95%CI was 1.321~6.028, *P* = 0.007), CRP ≥ 222.5 mg/L (OR = 3.298, 95% CI was 1.401–7.760, *P* = 0.006).

**Table 4:** Multivariate logistic regression analysis on the occurrence of septic shock.

**Variable**	**β value**	**S x̄**	**X2 value**	**OR value**	**95%CI**	***P*-value**
Highest HR ≥ 105 beats/min	1.091	0.383	8.101	2.977	1.405~6.311	0.004
Lac≥5.75 mmol/L	1.591	0.608	6.838	4.907	1.49~16.165	0.009
PCT≥41.47 ug/L	1.037	0.387	7.172	2.821	1.321~6.028	0.007
CRP≥222.5 mg/L	1.193	0.437	7.467	3.298	1.401~7.76	0.006
GCS score ≤ 14 points	0.914	0.405	5.082	2.494	1.127~5.522	0.024

HR, heart rate; GCS, Glasgow Coma Scale; Lac, blood lactate; PCT, procalcitonin; CRP, C-reactive protein; OR, odds ratio; 95% CI was 95% confidence interval.

### Determination of the score of each index

The regression coefficient of the β value obtained by Logistic regression analysis was assigned to calculate the ratio of the β value of the screened variables to the minimum β value and determine the score of the calculated ratio (Table 5). Finally, the clinical diagnostic score system of shock in patients with DTP was successfully constructed: the highest HR ≥ 105 beats /min (one point), GCS score ≤14 points (one point), Lac ≥ 5.75 mmol/L (2 points), PCT ≥ 41.47 ug/L (1 point), CRP ≥ 222.5 mg/L (one point), and the total score was 6 points.

**Table 5:** Coefficients and scores of diagnostic indicators of septic shock in patients with DTP.

**Indicator**	**β-value**	**Ratio**	**Score**
Highest HR ≥ 105 beats/min	1.091	1.194	1
Lac≥5.75 mmol/L	1.591	1.741	2
PCT≥41.47 ug/L	1.037	1.135	1
CRP≥222.5 mg/L	1.193	1.305	1
GCS score ≤14 points	0.914	1.000	1
Total score			6

HR, heart rate; GCS, Glasgow Coma Scale; Lac, blood lactate; PCT, procalcitonin; CRP, C-reactive protein; blank represented none.

### Diagnostic efficacy

**Figure 2:**
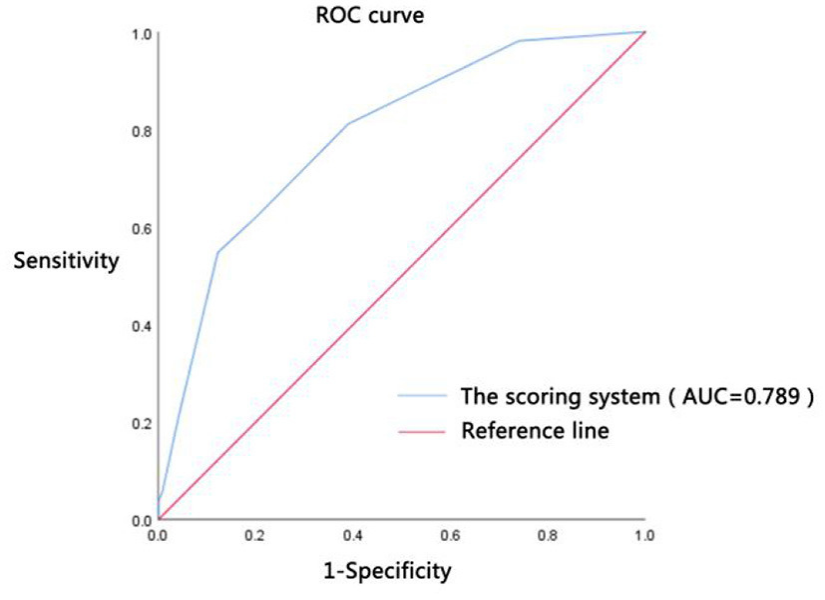
Diagnostic efficacy. ROC curve was the receiver operating characteristic curve; AUC was the area under the ROC curve; the scoring system was constructed by the highest HR ≥ 105 beats/min (1 point), Lac ≥ 5.75 mmol/L (two points), PCT ≥ 41.47 ug/L (one point), CRP ≥ 222.5 mg/L (one point), and GCS score ≤14 points (one point).

The scores of all patients were calculated according to the above-established scoring system for the clinical diagnosis of shock in patients with DTP; the clinical diagnostic value of shock was evaluated by the ROC curve (Figure 2). The results showed that the AUC of the scoring system for the septic shock diagnosis was 0.789, the 95% CI was 0.717–0.860 (*P* < 0.001). When the optimal cut-off value was 2.5 points, its sensitivity and specificity were 54.70 and 87.80%, respectively.

## Discussion

In this study, an early warning scoring system for septic shock in patients with DTP was successfully established based on general demographic data, perforation-related information, vital signs, common laboratory indicators, GCS score, APACHE-II score, and SOFA score. Early warning score of septic shock included: DTP = highest HR ≥ 105 beats/min (one point) + GCS score ≤14 points (one point) + Lac≥5.75 mmol/L (two points) + PCT ≥ 41.47 ug/L (one point)+CRP ≥ 222.5 mg/L (one point). When the optimal cut-off value was 2.5 points, the sensitivity and specificity were 54.70 and 87.80%, respectively. The early warning scoring system for septic shock in DTP will provide more possibilities for the treatment and diagnosis of patients with DTP.

Tachycardia is a warning sign of internal metabolic stress (23). Persistent tachycardia often suggests a poor prognosis in patients with septic shock (24). Songne *et al*. (25) found that HR > 94 beats/min was a significant predictor of failure of non-surgical treatment in patients with perforated peptic ulcers. Moreover, Møller et al. (26) suggested that tachycardia was one of the poor prognostic factors in patients with perforated peptic ulcers. Our study showed that the highest HR ≥ 105 beats/min was an independent risk factor for septic shock in patients with DTP, which was consistent with previous findings (24–26). Therefore, for patients with DTP, the HR should be closely monitored after admission.

Consciousness change is one of the three major clinical windows for assessing organ perfusion in patients with septic shock (27). Various scores associated with sepsis prognosis, including the SOFA score (28), APAPHE-II score (29), and the National Early Warning Score (NEWS) (30), have been used to assessing patient sanity with GCS score. Multiple studies have confirmed that GCS scores are associated with poor prognosis in patients with sepsis. In 1993, Basto et al. (31) found that lower GCS scores associated with sepsis were associated with higher mortality. A recent study by Wu et al. (32) confirmed that the GCS score was an important risk factor for predicting death in patients with sepsis. However, in the diagnostic criteria for septic shock proposed in Sepsis-3.0 (22), the GCS score is not a necessary condition for the diagnosis of septic shock but one of the detection items of SOFA score and has an auxiliary diagnostic value for septic shock. This study confirmed that a GCS score ≤14 points can be used as an independent risk factor for septic shock in patients with DTP, suggesting that consciousness change has a stronger early predictive value for patients with DTP. In addition, consciousness change was the manifestation of insufficient central perfusion in patients with sepsis, which was easily observed in clinical practice and had good timeliness and promotion. Therefore, compared with the diagnostic criteria of Sepsis-3.0 (22), the early warning scoring system of septic shock established in this study could assist clinicians in identifying patients with DTP combined with shock, thus guiding clinical diagnosis and treatment strategies.

Elevated arterial lactate is a manifestation of tissue hypoperfusion. In sepsis-3.0 (22), Lac ≥ 2.0 mmol/L is listed as one of the diagnostic criteria for septic shock. As an indicator of tissue hypoperfusion in patients with severe infection associated with patient prognosis, elevated lactate levels could assist clinicians in early predicting outcomes in patients with septic shock (33). In a retrospective study of 1,043 patients with septic shock, Oh et al. found a poorer prognosis in patients with high lactic acid compared to those with low lactic acid, suggesting that arterial lactic acid is a very reliable diagnostic and prognostic predictor of septic shock (34). Moreover, Bakker et al. (35) suggested that Lac >2 mmol/L is an independent risk factor for death in patients with septic shock. However, this study showed that Lac≥5.75 mmol/L was an independent risk factor for septic shock in patients with DTP, and Lac accounted for a high percentage of the early warning scoring system constructed in this study, suggesting that hyperlactatemia had a good early prediction value for septic shock in DTP patients. However, the optimal cut-off value of lactate in this study was significantly higher than the cut-off value of lactate in sepsis-3.0 (22), and the lactate level in the non-shock group was also significantly higher than the normal range which might be related to the combination of stress hyperlactatemia in patients with DTP. When the digestive tract is perforated, stress factors such as inflammation, pain, and surgical trauma could stimulate the secretion of catecholamines, leading to stress hyperlactatemia. Therefore, in a clinical setting, in addition to actively improving the microcirculation perfusion state, stress factors should also be actively controlled to reduce stress injury and avoid secondary injury caused by excessive resuscitation in patients with DTP complicated with hyperlactatemia.

As rapid and reliable markers of inflammation, serum procalcitonin (PCT) and C-reactive protein (CRP) play irreplaceable roles in diagnosing infectious diseases (36–38) and have a good clinical diagnosis and prognostic value for patients with sepsis and septic shock (39). A prospective study (40) of 78 patients with suspected sepsis admitted to the ICU suggested that PCT had good diagnostic and prognostic value in sepsis and septic shock. In a study of 423 patients with DTP, Grupp et al. (41) confirmed that elevated CRP had a predictive value for adverse outcomes in patients with DTP. In this study, we found that PCT ≥ 41.47 ug/L and CRP≥222.5 mg/L are independent risk factors for septic shock in patients with DTP, which further confirms that elevated levels of PCT and CRP could predict septic shock in patients with DTP. In this study, specific cut-off values of PCT and CRP levels were given, which were higher than those of other site infections. Therefore, it suggested that PCT and CRP might respond differently to infection at different sites, and enteric-borne infection might cause higher levels of PCT and CRP. In addition, it should be noted that since CRP was less specific, surgical trauma and other factors could also affect CRP levels, and reducing the interference of other factors on CRP might help obtain more valuable results.

This study has a few limitations. First, this study was a retrospective single-center study with relatively small sample size. Thus, a large-scale multi-center study is needed for further verification. Second, all patients included in this study were admitted to ICU, and those not admitted to ICU were excluded, which might lead to selection bias and affect the clinical characteristics of the non-shock group. Third, patients receiving antibiotics were not excluded in this study, which might affect the experimental results. However, since most patients with DTP in our hospital received emergency surgical treatment immediately after admission, the effect of antibiotic treatment on the experimental results should be relatively small based on the actual situation. Fourth, due to the limited sample size, this study has not been validated, which will limit the diagnostic efficiency and clinical application of the early warning scoring system. We will make further improvements in future larger studies.

In conclusion, the highest HR ≥ 105 beats/min, GCS score ≤14 points, Lac ≥ 5.75 mmol/L, PCT≥ 41.47 ug/L, CRP≥222.5 mg/L were independent risk factors for septic shock in patients with DTP. The early warning scoring system of septic shock in patients with DTP constructed based on these risk factors showed its certain clinical value, providing early warning indicators for clinicians to identify patients with DTP complicated with shock, which might improve the prognosis of patients. These indicators are easy to obtain in clinical practice and have been used in clinical practice for a long time, so they have a good promotion. The establishment of a scoring system based on common indicators may improve the prognosis of patients with septic shock in DTP, which is expected to contribute to the standardization of clinical teaching and practice of intensive care medicine and anesthesiology.

## Data availability statement

The raw data supporting the conclusions of this article will be made available by the authors, without undue reservation.

## Ethics statement

The studies involving human participants were reviewed and approved by Ethics Committee of Fujian Provincial Hospital, Fuzhou, China. The Ethics Committee waived the requirement of written informed consent for participation.

## Author contributions

PC and JG collected study data and drafted the present manuscript. XS revised the manuscript. All authors contributed to the article and approved the submitted version.

## Funding

The study was supported by Natural Science Foundation of Fujian [No. 2020J011086], Fujian Provincial Horizontal Issues grants [No. 2020-YC-001], and the high-level hospital foster grants from Fujian Provincial Hospital, Fujian Province, China [No. (2020) HSJJ14].

## Conflict of interest

The authors declare that the research was conducted in the absence of any commercial or financial relationships that could be construed as a potential conflict of interest.

## Publisher's note

All claims expressed in this article are solely those of the authors and do not necessarily represent those of their affiliated organizations, or those of the publisher, the editors and the reviewers. Any product that may be evaluated in this article, or claim that may be made by its manufacturer, is not guaranteed or endorsed by the publisher.
